# Addressing the Conflict between Mobility and Stability in Oxide Thin‐film Transistors

**DOI:** 10.1002/advs.202300373

**Published:** 2023-03-19

**Authors:** Lingyan Liang, Hengbo Zhang, Ting Li, Wanfa Li, Junhua Gao, Hongliang Zhang, Min Guo, Shangpeng Gao, Zirui He, Fengjuan Liu, Ce Ning, Hongtao Cao, Guangcai Yuan, Chuan Liu

**Affiliations:** ^1^ Laboratory of Advanced Nano Materials and Devices Ningbo Institute of Materials Technology and Engineering Chinese Academy of Sciences Ningbo 315201 P. R. China; ^2^ State Key Lab of Opto‐Electronic Materials and Technologies School of Electronics and Information Technology Sun Yat‐Sen University Guangzhou 510275 P. R. China; ^3^ Department of Materials Science Fudan University Shanghai 200433 P. R. China; ^4^ BOE Technology Group Co. Ltd. Beijing 100176 P. R. China; ^5^ Center of Materials Science and Optoelectronics Engineering University of Chinese Academy of Sciences Beijing 100049 P. R. China

**Keywords:** carrier relaxation, metal oxide semiconductors, percolation theory, photocarrier lifetime, thin‐film transistors

## Abstract

Amorphous oxide semiconductor thin‐film transistors (AOS TFTs) are ever‐increasingly utilized in displays. However, to bring high mobility and excellent stability together is a daunting challenge. Here, the carrier transport/relaxation bilayer stacked AOS TFTs are investigated to solve the mobility‐stability conflict. The charge transport layer (CTL) is made of amorphous In‐rich InSnZnO, which favors big average effective coordination number for all cations and more edge‐shared structures for better charge transport. Praseodymium‐doped InSnZnO is used as the charge relaxation layer (CRL), which substantially shortens the photoelectron lifetime as revealed by femtosecond transient absorption spectroscopy. The CTL and CRL with the thickness suitable for industrial production respectively afford minute potential barrier fluctuation for charge transport and fast relaxation for photo‐generated carriers, resulting in transistors with an ultrahigh mobility (75.5 cm^2^ V^−1^ s^−1^) and small negative‐bias‐illumination‐stress/positive‐bias‐temperature‐stress voltage shifts (−1.64/0.76 V). The design concept provides a promising route to address the mobility‐stability conflict for high‐end displays.

## Graphical abstract

A design concept of carrier transport/relaxation bilayer amorphous‐oxide‐semiconductor TFTs is demonstrated to avoid the bucket effect inevitable in single‐layer devices. In&Sn‐rich InSnZnO/InSnZnO:Pr bilayer TFTs that combine high mobility (75.5 cm^2^ V^−1^ s^−1^), robust normalized drain current (225 µA), and satisfactory NBIS/PBTS stability (voltage shift ∼ −1.64/0.76 V) are successfully developed.

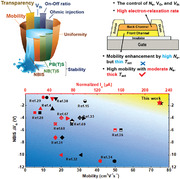

## 1 Introduction

Amorphous oxide semiconductor thin‐film transistors (AOS TFTs) serving as the pixel switch/drive devices have been widely used in the commercial flat panel displays (FPDs),^[^ ^1^ ^]^ because of their advantages of large‐area uniformity, low off‐state current, relatively high mobility, optical transparency, and so on.^[^ ^2^, ^3^, ^4^ ^]^ However, with the rapid development of information technology, the ever‐increasing demands of display technology elevate the requirements for TFTs. For example, the anticipated carrier mobility of TFTs used in a super hi‐vision (8 K) and ultrahigh frame rate (240 Hz) display is about 40 cm^2^ V^−1^s^−1^,^[^ ^5^ ^]^ whereas commercial In—Ga—Zn—O (IGZO)‐based TFTs are off specification.^[^ ^1^, ^6^ ^]^ The relatively low mobility also restricts the further development of novel display applications such as smart vehicles, distance learning, telemedicine, and augmented or virtual reality. Therefore, AOS TFTs with higher mobility are urgently needed for novel high‐end displays.

However, one cannot merely focus on the AOS TFTs’ mobility, other characteristics, such as threshold voltage (*V*_th_), on–off current ratio, uniformity, and stability, are equally important. For instance, the *V*_th_ of TFTs is expected to be close to but slightly higher than 0 V, so that TFTs can be switched on or off by a small voltage and the power consumption is at a minimum. Nevertheless, *V*_th_ would shift positively or negatively under some stress conditions, and the overly obvious threshold voltage shift (∆*V*_th_) does deteriorate the display quality. In a display panel, the switch TFTs are mostly in off‐state under illumination, and the off‐state duration will be longer in displays with higher resolution.^[^ ^1^, ^4^ ^]^ Unfortunately, the AOS TFTs generally have negative ∆*V*_th_ under negative‐bias illumination stress (NBIS) tests.^[^ ^7^, ^8^ ^]^ Although NBIS instability can be mitigated by using light‐shield layers, the introduced metal layer limits the improvement of refresh rate and aperture ratio of pixels. Meanwhile, TFTs are subjected to temperature rise when working. The trap states near the insulator/channel interface or/and in the gate insulator would be activated under the positive‐bias temperature stress (PBTS) tests, causing positive shift of *V*_th_.^[^ ^9^, ^10^, ^11^ ^]^ As for large‐area deposition, the uniformity of the active layer greatly affects the display module yield rate. In 8.5‐generation (G8.5) panel production line, the deterioration of uniformity would be serious if the active layer thickness is less than 20 nm. In brief, the evaluation of TFT performance is governed by the bucket effect as shown in Figure S1, Supporting Information, which reveals the status of G8.5 production line (2200 × 2500 mm) for high performance AOS TFTs. To prompt the display development, it is highly desirable that no obvious weakness is present in these characteristics. Most importantly, in ideal target devices, the stability will not deteriorate when the mobility increases. Here, we propose that the high‐mobility and illumination‐stability conflict can be circumvented by increasing indium content in AOS followed by forming a rapid recombination route for excess carriers. First, we deeply understand the intrinsic advantage of In‐rich AOS in charge transport and the deficiency in stability. Second, we compare several bilayer channel configurations and reveal the design rule most suitable to address the mobility‐stability conflict. We then examine the charge transport and carrier relaxation mechanisms that lead to the best device performance.

## 2 Results and Discussion

### 2.1 Leveraging In‐Rich Oxide Channels

**Figure 1:**
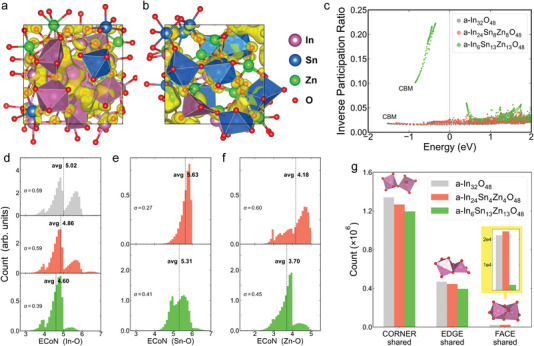
The partial charge density distribution of the lowest empty band for a) In_24_Sn_4_Zn_4_O_48_ and b) In_6_Sn_13_Zn_13_O_48_. Some of the metal‐centered coordination polyhedrons were also shown. c) Inverse participation ratio (IPR) of the conduction bands, where the fermi energy is set to zero. Average effective coordination number distributions of d) In, e) Sn, and f) Zn atoms. All these results were obtained as a statistical average of 5000 MD steps at room temperature and the same below. g) Quantities of the corner‐, edge‐, and face‐shared M—O polyhedron structures.

As the first step, enhanced mobility could be realized by bringing in more indium, which is quantitatively studied by theoretical calculations and experiments. We compare In_6_Sn_13_Zn_13_O_48_ and In_24_Sn_4_Zn_4_O_48_ (In‐rich) materials in amorphous structures which were generated using molecular dynamics (MD) simulations with melt‐and‐quench process. The simulated supercell was equilibrated at the room temperature for additional 5000 MD steps to sampling the amorphous structure and all structural properties (e.g., effective coordination number) were analyzed as a time average. The partial charge density distribution of the lowest empty band is shown in **Figure** **1**a,b, featuring some metal‐centered coordination polyhedrons. The electronic states are more extended in the amorphous In_24_Sn_4_Zn_4_O_48_ and tend to spread along the corner‐shared M—O polyhedral chain. Correspondingly, the inverse participation ratio (IPR) of a‐In_24_Zn_4_Sn_4_O_48_ near the conduction band minimum is much lower as compared with that in In_6_Zn_13_Sn_13_O_48_ (Figure 1c), signifying more delocalized electrons available. This result is related to the fact that the higher In content leads to the larger average effective coordination number (ECoN) of In—O bond in the amorphous oxides (Figure 1d–f), as found that the distribution of In—O bond ECoN in In_24_Sn_4_Zn_4_O_48_ is between five and six. For a reference, even in amorphous In_2_O_3_ the average ECoN of In—O is evidently smaller than the coordination number of six in the crystalline counterpart.^[^ ^12^ ^]^ Surprisingly, with more In content, the average ECoNs for Zn and Sn also increase toward the natural coordination numbers of Zn (4) and Sn (6), also benefiting the delocalized charge transport along the metal *n*s orbits. In addition, In‐rich In_24_Zn_4_Sn_4_O_48_ has more corner‐, edge‐, and face‐shared M—O polyhedrons as compared with In_6_Sn_13_Zn_13_O_48_ (Figure 1g). It has been verified that more corner‐, edge‐, and face‐shared polyhedron structures cause enhanced overlapping of electron wavefunction, in favor of high electron mobility.^[^ ^12^ ^]^ The calculated conduction band of a‐In_24_Sn_4_Zn_4_O_48_ is similar to In_2_O_3_ and more dispersive than that of a‐In_6_Sn_13_Zn_13_O_48_, indicating a small electron effective mass (Table S1, Supporting Information) to support a higher expected mobility. The MD calculations quantify why In‐rich AOS has better charge transport, which is compatible with the previous studies.^[^ ^2^, ^13^, ^14^, ^15^ ^]^ In experiments, we fabricated TFTs with In‐rich ITZO (In:Sn:Zn = 2:1:2) active layer or with ITZO (In:Sn:Zn = 1:2:2) and observed carrier mobility of 51.6 and 25.6 cm^2^ V^−1^ s^−1^ for them (Figure S2a, Supporting Information), respectively. The experimental results are consistent with the MD calculations.

However, In‐rich AOS itself in single‐layer channel AOS TFTs cannot achieve high mobility and strong stability.^[^ ^15^ ^]^ The experimental TFTs with In‐rich ITZO (In:Sn:Zn = 2:1:2) exhibit larger *V*_th_ shift in NBIS tests, as compared with those with ITZO (In:Sn:Zn = 1:2:2) (Figure S2b, Supporting Information). According to the first‐principle calculations, more indium atoms may induce a relatively narrow optical bandgap (*E*_g_), more oxygen vacancy (*V*_O_) defects acting as deep donor states,^[^ ^13^ ^]^ and more excess photo‐generated carriers under illumination. These effects, together with hydrogen‐related and carbon monoxide‐related impurities, lead to severe NBIS instability.^[^ ^15^, ^16^, ^17^, ^18^, ^19^ ^]^ Although doping foreign elements is feasible to improve it, for example, by Si,^[^ ^20^, ^21^, ^22^ ^]^ Ga,^[^ ^16^ ^]^ Hf,^[^ ^17^, ^23^ ^]^ Zr,^[^ ^24^ ^]^ and Pr,^[^ ^25^, ^26^ ^]^ the sacrifice of mobility as well as PBTS stability is inevitable.^[^ ^23^, ^24^, ^27^ ^]^

### 2.2 Relaxing Photo‐Electrons in Bilayer Channel

**Figure 2:**
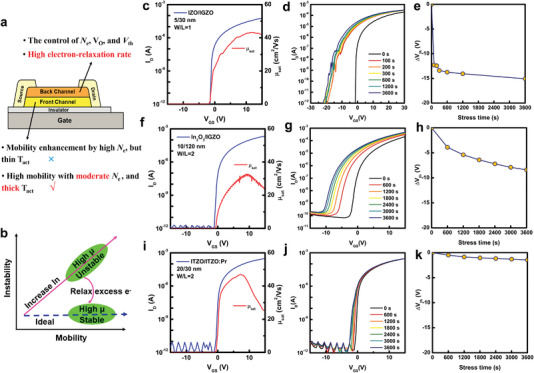
Bilayer transistor characteristics of various material combinations. a) Design concept of conventional and novel bilayer channel. b) A strategy to address the mobility‐stability conflict to reach target devices. Transfer characteristics (*I*_D_–*V*_GS_) and time‐dependent ∆*V*_th_ under NBIS tests (*V*_GS_ stress = −20 V, 3600 s, halogen light power density = 0.22 mW cm^−2^) of c–e) IZO/IGZO, f–h) In_2_O_3_/IGZO, and i–k) ITZO/ITZO:Pr bilayer TFTs.

Splitting the single‐layer channel into stacked bilayer is an operable scheme^[^ ^4^, ^21^, ^28^, ^29^, ^30^, ^31^, ^32^, ^33^, ^34^, ^35^, ^36^ ^]^ (**Figure** **2**a). Usually, the front channel is with high electron concentration (*N*_e_) for transporting carriers,^[^ ^37^, ^38^, ^39^, ^40^, ^41^, ^42^ ^]^ while the back channel layer has low *N*_e_ to control *V*_th_, to suppress *V*_O_, and to balance the overall *N*_e_.^[^ ^43^, ^44^, ^45^ ^]^ As examples, we fabricated InZnO/InGaZnO (IZO/IGZO) and In_2_O_3_/IGZO bilayer channel TFTs and achieved relatively high mobility (>40 cm^2^ V^−1^ s^−1^, Figure 2c,f). However, there are still some close concerns: the thickness of the front‐channel layer is usually below 10 nm to avoid negative *V*_th_,^[^ ^33^, ^36^, ^37^, ^46^, ^47^, ^48^ ^]^ creating difficulties in industrial, large‐area manufacturing; also, the NBIS stability issues are still pending, whether for our attempts (Figure 2d,e,g,h) or for the reports in the literatures.^[^ ^32^, ^33^, ^34^, ^35^, ^36^ ^]^

To hold high mobility and NBIS stability together, we propose another design strategy, that is, increasing indium content in AOS and providing a rapid recombination route for excess carriers (Figure 2b). Here, we use bottom‐gate, top contact device configuration with SiO_2_/Si substrates for the proof of concept, similar to the previous works.^[^ ^15^, ^49^ ^]^ Unlike conventional bilayer structure, AOS films with moderate *N*_e_ and high mobility are set as the front channel, whereas AOS films with rapid electron‐relaxation rate are used as the back channel. As proof of concept, In‐rich ITZO (In:Sn:Zn = 2:1:2) was deposited under high oxygen partial pressure at the front channel to serve as carrier transport layer (CTL, *N*_e_ ≈ 3.64 × 10^16^ cm^−3^), while praseodymium‐doped ITZO (In:Sn:Zn:Pr = 1:2:2:0.17) is set at the back channel as carrier relaxation layer (CRL). According to the Aladdin list, the price of 100 g Pr_6_O_11_ is about 24% of 100 g In_2_O_3_ (both with 4N purity), so the material cost caused by using rare earth element is acceptable. By doping a small amount of Pr, more importantly, the photoelectron lifetime is shortened, contributing to the NBIS stabilization.^[^ ^25^, ^26^, ^27^ ^]^ Also, the ITZO semiconductors possessing high resistance to acids and alkalis can help to realize both back‐channel‐etch and etch‐stop‐layer type TFTs,^[^ ^1^, ^37^, ^47^ ^]^ and the similar chemical composition between the CTL and CRL benefits the interface quality and wet etching process. As shown in Figure 2i–k, the optimized devices present a small *V*_th_, relatively high mobility of 46.8 cm^2^ V^−1^ s^−1^, and small NBIS ∆*V*_th_ of −1.56 V, which is much better than those designed by the conventional strategy (Figure 2c–h). In particular, the film thickness of the proposed devices is above 20 nm for both the CTL and CRL, which is compatible with large‐area production for display panels. Therefore, the design strategy, increasing In‐content and adding photo‐carrier relaxation path (Figure 2b), provides a practical design rule for solving the “bucket effect” problem that limits the development of high‐performance TFT manufacturing (Figure S1, Supporting Information).

**Figure 3:**
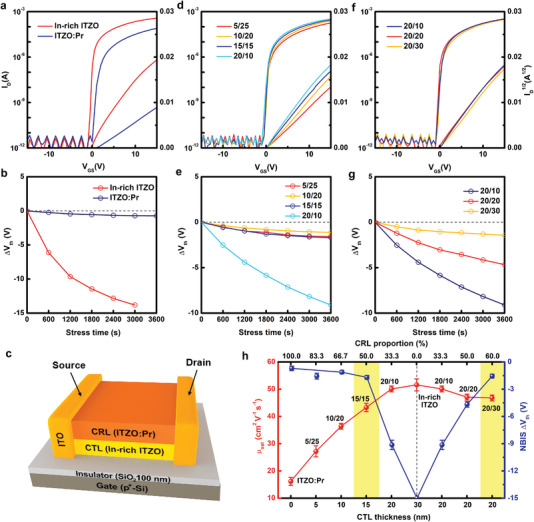
Electrical performance of single‐layer and bilayer TFTs. a) Transfer characteristics and b) time‐dependent NBIS ∆*V*_th_ of 30 nm‐thick single‐layer In‐rich ITZO and ITZO:Pr TFTs. c) Schematic diagram of the CTL/CRL bilayer TFTs. Transfer characteristics and time‐dependent NBIS ∆*V*_th_ of d,e) 30 nm‐thick bilayer TFTs and f,g) bilayer TFTs with 20 nm‐thick CTL and varied CRL. h) The *µ*_sat_ and NBIS ∆*V*_th_ extracted from the corresponding TFTs. The error bars represent the standard deviation over 15 devices and devices with high *µ*_sat_ and good NBIS stability are marked inside the yellow color zone.

The bilayer TFTs were systematically investigated. First, the single layer In‐rich ITZO and ITZO:Pr TFTs are characterized in **Figure** **3**a. In‐rich ITZO TFTs possess high saturation mobility (*µ*_sat_) (51.6 cm^2^ V^−1^ s^−1^) but the ∆*V*_th_ under NBIS tests is beyond −15 V (Figure 3b and details in Figure S3, Supporting Information). The NBIS instability is closely associated with light‐induced ionization effect, since the NBS stability of In‐rich ITZO TFTs is pretty good (∆*V*_th_ = −0.28 V, Figure S4, Supporting Information). By contrast, ITZO:Pr TFTs have *µ*_sat_ of 16.2 cm^2^ V^−1^ s^−1^ and a small ∆*V*_th_ of −0.71 V under NBIS (Table S2, Supporting Information). In CTL/CRL bilayer structure TFTs (Figure 3c), we fabricated devices with the same total channel thickness but with different thickness ratios (Figure 3d,e), and devices with the same CTL thickness but with varied CRL thickness (Figure 3f,g), respectively. The top contact device configuration is chosen for the simplicity in checking the interface. We use unpatterned SiO_2_/Si substrates for the gate dielectric and gate electrodes for the simplicity to focus on the CTL/CRL interface. With fixed total thickness, as the CTL/CRL ratio increases (CTL/CRL = 5/25, 10/20, 15/15, and 20/10 nm), the on‐current in the transfer (Figure 3d) and output (Figure S5, Supporting Information) characteristics increases and the *µ*_sat_ is boosted up from 27.2 to 50.1 cm^2^ V^−1^ s^−1^. Although the ∆*V*_th_ in NBS tests is small (approximately −0.10 V, Figure S4, Supporting Information), the ∆*V*_th_ in NBIS test varies substantially from −1.56, −1.12, −1.70, to −9.13 V for the 5/25, 10/20, 15/15, and 20/10 devices (Figure 3e and Figure S3, Supporting Information). For another comparison, CTL thickness was kept at 20 nm to ensure high *µ*_sat_, while CRL thickness was varied (CTL/CRL = 20/10, 20/20, and 20/30 nm). The transfer and output characteristics are displayed in Figure 3f and Figure S5, Supporting Information. The *µ*_sat_ is decreased slightly from 50.1 to 46.8 cm^2^ V^−1^ s^−1^ but the NBIS ∆*V*_th_ is dramatically suppressed from −9.13 (20/10) through −4.66 (20/20) to −1.56 V (20/30) (Figure 3g and Figure S3, Supporting Information). These results are summarized in Figure 3h, indicating that 20 nm CTL is enough to afford high mobility and CRL slightly thicker than CTL is required to ensure good NBIS stability.

The time‐dependent ∆*V*_th_ under PBTS tests is exhibited in Figures S6 and S7, Supporting Information. It is found that the PBTS ∆*V*_th_ is highly dependent not on the CRL but the CTL thickness, similar to the *µ*_sat_ variations. The bilayer TFTs with 20 nm‐thick CTL have displayed sound PBTS stability and *µ*_sat_ that are comparable to those of single‐layer In‐rich ITZO TFTs, suggesting that high mobility and good PBTS stability can be successfully obtained by employing In‐rich and thick‐enough CTL. More device performance details are shown in Figures S3–S7, Supporting Information and are summarized in Table S1, Supporting Information.

### 2.3 Probing Interface and Electronic Structure

**Figure 4:**
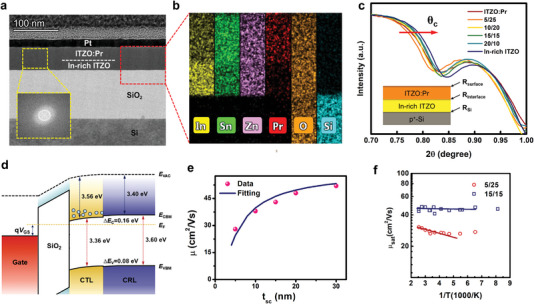
Mobility enhancement mechanism in indium‐rich ITZO/ITZO:Pr bilayer TFTs. a) Cross‐sectional HR‐TEM image and b) EDS mapping results of bilayer TFTs (ITZO/ITZO:Pr = 20/30). The inset to (a) shows the fast Fourier transformed image of the bilayer AOS film. c) Enlarged graph of XRR spectra, *θ*_c_ represents the critical angle for total external reflection. The inset shows the fitting model for roughness in XRR. d) Energy‐level alignment diagram of indium‐rich ITZO and ITZO:Pr. e) Thickness‐dependent mobility of bilayer channel transistors, and *t*_sc_ represents the film thickness of the CTL. f) Temperature‐dependent mobility of bilayer transistors.

The bilayer channel interface is examined as field effect mobility is associated with the interface scattering. The high‐resolution cross‐sectional transmission electron microscope (HR‐TEM, **Figure** **4**a) of a representative sample (20/30) shows an amorphous nature of the whole bilayer. This is also consistent with the Fast Fourier Transformed result (the insert of Figure 4a) which shows diffused hollow rings corresponding to the amorphous phase. Also, the undistinguishable interface is inferred from the HR‐TEM picture, because the bilayer films are the same ITZO‐based materials. The energy dispersive spectrometer (EDS) mapping for the In, Sn, and Pr element demonstrates clear interface without element diffusion between the two layers (Figure 4b). The X‐ray reflectometry (XRR, Figure 4c; Figure S8 and Table S3, Supporting Information) reveals that the In‐rich ITZO surface (In‐rich ITZO/air, *R*_surface_ = 0.64 nm), ITZO:Pr surface (ITZO:Pr/air, *R*_surface_ = 0.42–0.53 nm), and bilayer interface (In‐rich ITZO/ITZO:Pr, *R*_interface_ = 0.49–0.55 nm) are all very smooth, guaranteeing minimal interface scattering. From the XRR spectra, the effective density (*ρ*_eff_) of the films was extracted at the critical angle (*θ*_c_) (Figure 4c and Table S3, Supporting Information), as larger *θ*_c_ implies bigger *ρ*_eff_.^[^ ^50^, ^51^ ^]^ With increasing the thickness ratio of CTL/CRL, the *ρ*_eff_ of bilayer films varies monotonously between 8.12 and 8.52 g cm^−3^, except the 5/25 bilayer channel with a relatively small *ρ*_eff_ (7.97 g cm^−3^). This suggests that ultrathin front‐channel leads to poor uniformity and small film packing density, which explains why 5/25 bilayer TFTs exhibit poor NBIS stability (Figure 3h). Generally, the stacked bilayers have abrupt and smooth interface, implying that the charge transport is not limited by the interface scattering.

The energy structures of the bilayer film are examined by spectroscopic ellipsometry and ultraviolet photoelectron spectroscopy (UPS) measurement. The extracted energy‐level alignment diagram is demonstrated in Figure 4d. The *E*_g_ of ITZO:Pr (3.60 eV) is slightly larger than that of In‐rich ITZO (3.36 eV), as the *E*_g_ of In_2_O_3_ is about 2.9 eV^[^ ^52^ ^]^ and PrO*_x_* is about 3.9 eV.^[^ ^53^ ^]^ Details of the energy gap between Fermi level and valence‐band minimum (*E*_F_ *− E*_VBM_) and work function (*Φ*_F_) are described in Figure S9, Supporting Information. Importantly, the conduction band offset (∆*E*_C_) between In‐rich ITZO and ITZO:Pr is as small as 0.15 eV, resulting in negligible potential barrier for electron transfer between them. Therefore, under positive *V*_g_, the downward band bending of *E*_CBM_ occurs together with intensive electron accumulation and the *µ*_sat_ is primarily influenced by the electron transport within CTL.^[^ ^54^, ^55^ ^]^ Under negative *V*_g_, on the other hand, the upward band bending of *E*_CBM_ occurs together with electron depletion, so the photo‐generated carriers in CTL can easily diffuse into CRL owing to the small potential barrier between them.

### 2.4 Demystifying the Mechanisms for Transport and Stability

Since carrier transport under positive *V*_g_ is within CTL, the relation between mobility and the CTL thickness (Figure 4e) can be accounted by the percolation theory. The theory assumes that current is through a lattice in which there are similar resistances that join the neighboring sites, whereas the broken bonds or other deep defect states correspond to infinite resistance.^[^ ^56^ ^]^ The charge hopping possibility increases with the charge transport path, which increases exponentially with *t*_sc_ if the film is very thin but saturates if the film is thick. The carrier mobility follows^[^ ^57^, ^58^ ^]^

(1) $$\mu \;\left(t_{\mathrm{sc}}\right)=\;\mu^{\prime }\exp\left\{-\xi_{0}\left[1+{\left(\frac{t_{\mathrm{sc}0}}{t_{\mathrm{sc}}}\right)}^{1/\nu }\right]\right\}$$

where *μ*′ is the characteristic mobility, *ξ*_0_ the dimensionless percolation threshold, *t*_sc0_ the characteristic film thickness, and *ν* the 3D index of the correlation radius (≈0.9).^[^ ^59^, ^60^ ^]^ The experimental data of saturated mobility are well fitted by using the parameter of *t*_sc0_ = 25 nm, the percolation threshold = 0.15, and *ν* = 0.9. In terms of temperature‐dependent measurements, as a potential fluctuation is formed above the mobility edge, the distribution of the potential barrier height *ϕ* causes distinct electron transport behaviors and temperature‐activated mobility is^[^ ^60^ ^]^

(2) $$\mu \;\left(T\right)=\;\mu^{\prime \prime }\exp\left(-\frac{\phi_{0}-q\sigma_{\phi }^{2}/2kT}{kT/q}\right)$$

where *ϕ*_0_ and *σ*_*ϕ*_ is the average and the distribution variance of the potential barriers, respectively, and *μ*′′ the characteristic mobility. If *σ*_*ϕ*_ = 0, Equation (2) turns to be the Arrhenius form for activating electrons from a single‐level trap state and *ϕ*_0_ represents the activation energy. Transistors with two representative thickness ratios (5/25 and 15/15) are studied and the results are shown in Figure 4f. The *μ*(*T*) exhibits thermal‐activated features as it decreases with *T* (i.e., $\phi_{0}-q\sigma_{\phi }^{2}/2kT>0$) in the transistor (5/25 thickness ratio), whereas the *μ*(*T*) is almost temperature‐independent (i.e., $\phi_{0}-q\sigma_{\phi }^{2}/2kT\approx 0$) in the other one (15/15 thickness ratio). This is highly consistent with the above finding that thicker In‐rich ITZO films as CTL in the bilayer structured TFTs have minute potential barrier fluctuation, in favor of charge transport. The experimental and theoretical fitting results indicate that 20 nm thick In‐rich ITZO film forms a promising CTL in the bilayer structured TFTs, meeting the requirements of mobility, PBTS stability, and large‐area uniformity.

**Figure 5:**
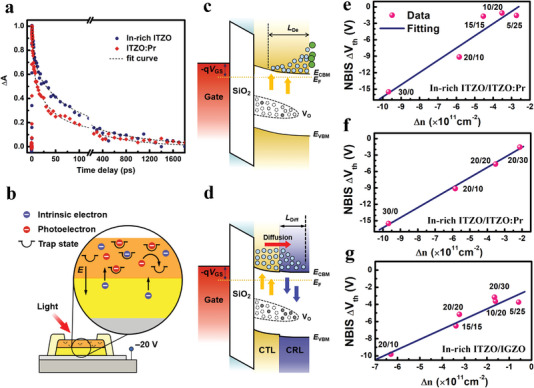
Stability enhancement mechanism of CTL/CRL bilayer TFTs. a) Normalized transient absorption (∆*A*) decay dynamics of In‐rich ITZO and ITZO:Pr films characterized by femtosecond TAS. b) The design concept of bilayer AOS TFTs to achieve both high mobility and NBIS stability. The schematic diagram of the generation, diffusion, and recombination of photo‐induced electrons in c) the single‐CTL and d) bilayer TFTs. c,f) Relationship between the NBIS ∆*V*_th_ and the predicted excess carrier density ∆*n* for the In‐rich ITZO/ITZO:Pr TFTs. g) The ∆*V*_th_ versus ∆*n* for the In‐rich ITZO/IGZO TFTs.

The NBIS stability issue is studied based on the photoelectron relaxation measurements. The photoelectron lifetime is characterized by femtosecond transient absorption spectroscopy (TAS, **Figure** **5**a). The multi‐exponential decay curves can be fitted by a tri‐exponential decay model^[^ ^61^ ^]^ $\Delta A\left(t\right)=\sum_{i=1,2,3}A_{i}\exp\left(-\frac{t}{\tau_{i}}\right)$, where ∆*A* is the normalized transient absorption, *A*_i_ the weight of the relaxation process, and *τ*_i_ the lifetime of photoelectrons. As a rule of thumb, *τ*_1_, *τ*_2_, and *τ*_3_ lasts for 0–10, 10–100, and >100 ps, respectively relating to the redistribution of photoelectrons among shallow trap states, non‐radiative relaxation of electrons toward deeper trap states, and the recombination of electrons from trap states to the ground ones.^[^ ^61^ ^]^ The photoelectron lifetime in ITZO:Pr is approximately halved than that of ITZO in all timescales (Table S4, Supporting Information), accelerating the process from the excited states to the equilibrium ones for photoelectrons in the CRL and contributing to small ∆*V*_th_ of TFTs under NBIS tests.

The schematic diagram of the NBIS stability mechanism is illustrated in Figure 5b–d. In TFTs with single layer CTL (e.g., ITZO), both the effects of photo‐generation and self‐passivation are considered (Figure 5c). For photo‐generation, excess carriers are photo‐generated with a generation rate *G* and a mean life‐time *τ*, giving the total photo‐generated carrier density at quasi‐steady state as $\Delta \;n_{\mathrm{bulk}}=\;G\tau_{1}\frac{t_{\mathrm{CTL}}}{t_{0}}$, where the term $\frac{t_{\mathrm{CTL}}}{t_{0}}$ describes the photo‐generated carriers that increase with the CTL film thickness *t*_CTL_ and decrease with *t*_0_ (a characteristic thickness constant).^[^ ^62^ ^]^ For self‐passivation, the charged ions adsorbed at the back‐channel surface with the density *N*_surf_ induce the increased carrier density in the semiconductors, which can be characterized by the Debye length *L*_De_.^[^ ^63^, ^64^ ^]^ Therefore, for a single layer ITZO TFT, the excess carrier density causing the threshold voltage shift at the quasi‐steady state is

(3) $$\begin{array}{ccc} \Delta n_{\mathrm{single}} & = & \;G\tau_{1}\frac{t_{\mathrm{CTL}}}{t_{0}}+N_{\mathrm{surf}}\exp\;\left(-\frac{t_{\mathrm{CTL}}}{L_{De}}\right)\\ & = & \;\Delta n_{\mathrm{bulk}}\left[1+a\exp\left(-\frac{t_{\mathrm{CTL}}}{L_{\mathrm{De}}}\right)\right] \end{array}$$

where $a\;=\frac{N_{\mathrm{surf}}}{\Delta n_{\mathrm{bulk}}}\;$. As the *L*_De_ for ITZO film in this study is calculated to be 21.8 nm, the values of Δ*n*_single_ can be calculated and compared with the experimentally measured Δ*V*_th_. In bilayer channel, the photo‐generated carriers in the CTL easily diffuse into the CRL (the conduction band offset between them is only 0.15 eV) and then recombine, whereas the self‐passivation effect in single layer case can be ignored (Figure 5d). Therefore, the residue excess carriers are^[^ ^62^ ^]^

(4) $$\Delta n_{\mathrm{bilayer}}=\;G\tau_{1}\frac{t_{\mathrm{CTL}}}{t_{0}}\;\times \;\exp\left(-\frac{t_{\mathrm{CRL}}}{L_{\mathrm{Diff}}}\right)$$

The first term has been obtained in single layer CTL transistor and the second term characterizes the diffusive recombination in CRL. The diffusion length for CRL (ITZO:Pr) is estimated by $L_{\mathrm{Diff}}=\sqrt{D_{\mathrm{CRL}}\tau_{\mathrm{CRL}}}\;$(19.7 nm), using the mean‐free time *τ*_CRL_ obtained by the transient absorption decay dynamics experiments (*τ*_CRL_ = 30 ps) and the diffusion coefficient *D*_CRL_ estimated by mobility of TFTs with single CRL ($D_{\mathrm{CRL}}\approx \frac{kT}{q}\mu \;=\;0.13$ cm^2^ s^−1^). The predicted Δ*n*_bilayer_ is in good linear relation with the experimentally extracted Δ*V*_th_ as shown in Figure 5e,f. In general, the theory verification echoes very well with our experimental results (Figure 3 and Table S2, Supporting Information), verifying that the strategy using rapid photoelectron relaxation CRL thicker than its diffusion length *L*_Diff_ (≈20 nm) is capable of resolving NBIS difficulty. This deduction is also well consistent with the variation of ionized *V*_O_ whose number is a determining factor for the device's instability. In the bilayer structure, the photo‐induced electrons in the CTL quickly move into the CRL, and then can promote the neutralization of ionized *V*_O_ with aid of the Pr dopant in the CRL.^[^ ^65^, ^66^ ^]^ Consequently, the total number of ionized *V*_O_ in the bilayer decreases, leading to reduced photocurrent of the bilayer TFTs. It may be an average effect. Hence, the CRL proportion is very important for the NBIS stability of the bilayer devices (Figure 3h), as analyzed from the relationship between the photoelectron variations and NBIS ∆*V*_th_ (Figure 5e,f).

The strategy is further examined by using inverted structures or changing specific materials. In the reverse‐bilayer‐structure TFTs, CRL and CTL are respectively set as the front‐ and back‐channel. With the CRL/CTL ratio varying from 10/20, 20/20, to 30/20 nm, the reverse‐bilayer TFTs has much lower *µ*_sat_ inferior to that of the counterparts and poor PBTS (|∆*V*_th_| > 5 V) and NBIS (|∆*V*_th_| > 15 V) stability. The electrical performance is shown in Figure S10 and Table S5, Supporting Information. Therefore, the stacking order cannot be inverted in bilayer AOS TFTs. The design concept is further checked for general applicability. Instead of ITZO:Pr, Ga‐rich IGZO (In:Ga:Zn = 1:2:1) was employed as CRL to build In‐rich ITZO/IGZO bilayer TFTs. With changing CTL and CRL thickness, the mobility and stability of In‐rich ITZO/IGZO bilayer TFTs exhibit almost the same trend as the In‐rich ITZO/ITZO:Pr devices. The detailed information can be referred to Figure S11 and Table S6, Supporting Information. Remarkably, the predicted Δ*n* is also in good linear relation with the experimentally extracted ∆*V*_th_ for the In‐rich ITZO/IGZO bilayer TFTs (Figure 5g). These results confirm the general applicability of the proposed design concept, and one should carefully select both CTL and CRL materials in terms of their labeled physical properties.

### 2.5 Achieving Ultrastable Bilayer TFTs with High Mobility of 75.5 cm^2^ V^−1^ s^−1^

**Figure 6:**
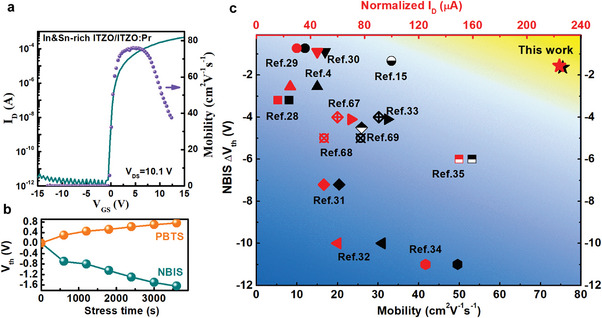
Electrical properties of In and Sn‐rich ITZO/ITZO:Pr bilayer TFTs. a) Transfer characteristics and b) time‐dependent ∆*V*_th_ under NBIS and PBTS tests. c) Comparison of mobility, normalized *I*_D_, and NBIS stability of a variety of bilayer AOS TFTs from the literature and this work. The symbols in red color represent the normalized *I*_D_ and the black ones represent the mobility. The normalized *I*_D_ is the saturation drain current *I*_D,sat_ divided by *W*/*L* (*I*_D,sat_ corresponds to the drain current at *V*_GS_ = 15 V × *t*_die_/100 nm, where *t*_die_ is the thickness of SiO_2_ and SiN*_x_*). In some literature, the semiconductor patterns are wider than the electrodes and thus the channel width *W* was used to extract the normalized *I*_D._ If the channel is not probably patterned, the mobility is divided by the correction factor to consider the contribution of the fringe‐current.^[^ ^15^ ^]^

Based on the above‐proposed design concept, we further explore ITZO rich of both In and Sn (In:Sn:Zn = 3:2:1) as the CTL to elevate the mobility. The orbitals of Sn 5s and In 5s show larger overlapping parts compared to Zn 4s, the synergic intercalation between which is expected to widen the electron transport path and improves the mobility. The In and Sn‐rich single layer TFTs demonstrate an ultrahigh mobility of around 80.5 cm^2^ V^−1^ s^−1^ (Figure S12, Supporting Information) and the corresponding In and Sn‐rich ITZO/ITZO:Pr (20/30 nm) bilayer TFTs present an ultra‐high mobility of 75.5 cm^2^ V^−1^ s^−1^ (**Figure** **6**a), and small NBIS ∆*V*_th_ of −1.64 V and PBTS ∆*V*_th_ of 0.76 V (Figure 6b and details in Figure S13a, Supporting Information), which is the best one among the literatures (Figure 6c and details in Table S7, Supporting Information)^[4,15,28–35,67‐69]^. We compare the charge transport properties and illumination stability in terms of mobility and NBIS threshold shift of our devices with other reported transistors, including single‐layer and bilayer channels deposited by sputtering. As shown in Figure 6c, some transistors achieve very high mobility (beyond 30 cm^2^ V^−1^ s^−1^) but the NBIS threshold shift exceeds −4 V,^[32‐35]^ whereas those very stable transistors with the NBIS threshold shift less than −1 V exhibit the mobility less than 20 cm^2^ V^−1^ s^−1^.^[29,30]^ Notice that the normalized *I*_D_ is used to conduct cross‐group comparison, in order to get convincing and fair results. The normalized *I*_D_ of our devices is 225 µA (as also depicted by the output curves in Figure S13b, Supporting Information), quite competitive among the literature. This illustrates that the proposed TFT is able to well balance the mobility and illumination stability, as supported by that the current is in weak response to the green and blue light (450 nm) (Figure S14, Supporting Information). The NBIS stability of our TFTs is comparable to that of IGZO TFTs (Table S6, Supporting Information), but the *µ*_sat_ is more than six times higher.

## 3 Conclusion

In‐rich ITZO/ITZO:Pr bilayer TFTs that combine high mobility (*µ*_sat_ = 75.5 cm^2^ V^−1^ s^−1^), robust normalized drain current (225 µA), and satisfactory NBIS/PBTS stability (∆*V*_th_ = −1.64/0.76 V) have been successfully developed based on the design concept of CTL/CRL structured channel. After comprehensive investigations, we find out the code for addressing the mobility‐stability conflict. To gain high mobility, the indium‐rich CTL located at front‐channel region, featuring large average M—O effective coordination numbers, enhanced overlapping of electron wavefunction, and minute potential barrier fluctuation in nature, has the ability to produce high electron mobility. Also, the thickness of CTL no less than 20 nm allows large‐area uniformity deposition, facilitating the build of high‐class gate insulator/channel interface to support sound PBTS stability. Then, to achieve good NBIS stability, excess photo‐generated carriers are relaxed by using praseodymium‐doped ITZO film as CRL to cut down the photoelectron lifetime. The CRL thicker than its carrier diffusion length helps to achieve favorable NBIS stability. Based on the theoretical and experimental work, the design concept is generally applicable to break the deadlock on how to combine the mobility and stability together in AOS TFTs, thereby earning future opportunities for the real applications of upgraded AOS TFTs in high‐end displays.

## 4 Experimental Section

### Calculation Method

All calculations were based on the plane wave pseudopotential method within the density functional theory. The projector augmented wave method was used as implemented in the Vienna ab initio simulation package.^[^ ^70^, ^71^ ^]^

The amorphous structures were obtained using molecular dynamics (MD) simulations. The crystalline conventional cell with 32 metal atoms was melted at 3000 K for 30 ps, then quenched to 100 K with a cooling rate of 200 K p^−1^ s^−1^, and equilibrated at 300 K for 6 ps. To overcome the limitation of representing the amorphous structure using atoms in a single cell, the structures were equilibrated at room temperature for additional 5000 MD steps, and all structural properties (e.g., effective coordination number and pair correlation function) were analyzed as a time average. It shows that the period of 5000 steps was long enough for these results to reach convergence. The MD simulations were carried out in the NVT ensemble using an integration time step of 3 ps, and the PBEsol functional was employed to describe the exchange–correlation interactions. The first Brillouin zone was sampled with a single‐Γ point. The cut‐off energy was set to 400 eV during melting and quenching, and to 420 eV during the equilibration. The melting process was performed with an on‐the‐fly machine learning force field MD^[^ ^72^ ^]^ to speed up the calculation, and other ones were performed with ab‐initio MD.

Geometry optimization was carried out using the SCAN meta‐GGA functional.^[^ ^73^ ^]^ The electronic structure was calculated with the modified Becke–Johnson (MBJ) meta‐GGA functional, from which the band structure, charge density distribution, and IPR were obtained. The IPR of an orbital *ψ_n_* (*r_i_*) was calculated by^[^ ^74^ ^]^

(5) $$\mathrm{IPR}\;\left(\psi_{n}\right)=\frac{\sum_{i=1}^{N}{\left|\psi_{n}\left(r_{i}\right)\right|}^{4}}{{\left(\sum_{i=1}^{N}{\left|\psi_{n}\left(r_{i}\right)\right|}^{2}\right)}^{2}}\;$$

where *N* is the number of atoms. Note that, the definition of IPR may vary by a constant *N*. Here, IPR was in the range of 1/*N* to 1. During these calculations, the cut‐off energy was chosen to be 450 eV, and a 2 × 2 × 2 Γ‐centered k‐point mesh was selected. The atomic configurations and charge density distribution were visualized using VESTA.^[^ ^75^ ^]^

### Device Fabrication

Bottom gate structure TFTs were fabricated on the heavily doped silicon substrate with a 100 nm‐thick thermal SiO_2_ layer, serving as the gate electrode and insulator, respectively. In‐rich ITZO films were deposited by direct‐current magnetron sputtering with the power of 80 W and the high oxygen partial pressure of O_2_/(Ar + O_2_) = 40%. And ITZO:Pr films were deposited by radio‐frequency magnetron sputtering with the power of 80 W and the high oxygen partial pressure of O_2_/(Ar + O_2_) = 50%. After deposition, all the films were annealed at 350 °C in the air atmosphere for 1 h. For source (S) and drain (D) electrodes, 100 nm‐thick In—Sn—O (ITO) films were deposited by direct‐current magnetron sputtering. Post‐annealing (150 °C for 1 h) was performed to improve electrode contact and recover damage from electrode sputtering. Both the channel layer and S/D electrodes were patterned by shadow masks. The width/length (*W*/*L*) of the TFTs was 800/400 µm. A top‐view micrograph of the fabricated devices is shown in Figure S15, Supporting Information. For these devices, the S/D electrode strips were 100 µm longer than the *W* of the channel layer at both ends. Therefore, the mobility extraction was completely free of the edge effect.

### Material Characterization

The absorption coefficient (*α*) of films were measured by variable angle spectroscopic ellipsometry (SE, M‐2000 DI, J. A. Woollam). The thickness of each layer in the bilayers was determined from accurate fitting of the obtained ellipsometric data using the software WVASE. The TEM image of the 20/30 bilayer film was also used for calibration. The thickness error by the SE method was within 1 nm. The roughness and effective density (*ρ*_eff_) of films were characterized by XRR (D8 DISCOVER, Bruker). All samples were modeled as simple stacked films. The microstructure of the films was analyzed by HR‐TEM (Talos F200X, Themo Fisher) with EDS. The band structure of the films was determined by UPS (Axis Ultra DLD, Kratos) and the *E*_g_ extracted from the horizontal intercept of the linear part in Tauc plot (the square of the product of the *α* and photon energy, (*αhυ*)^2^, vs photon energy (*hυ*)).

The relative permittivity (*ε*_r_) of In‐rich ITZO and ITZO:Pr was extracted in the capacitance–voltage (*C*–*V*) curves of the metal–insulator–semiconductor (MIS) capacitors analyzed by precision LCR meter (E4980A, Agilent) at 1 kHz, as shown in Figure S16, Supporting Information. The stacked MIS samples were fabricated in the same process as TFTs. The electron concentration (*N*_e_) of films was tested by Hall‐effect measurements (8400 series, Lakeshore) with conventional van der Pauw configuration. The films were deposited on 1 × 1 cm^2^ quartz substrate and 100 nm‐thick ITO films were deposited at the four corners of the Hall sample to achieve Ohmic contacts.

The femtosecond TAS was measured with an amplified Yb:KGW femtosecond laser (1.20 eV, 190 fs, and 6 kHz) a standard white‐light pump‐probe spectroscopy. The pump photon energy was fixed at 3.49 eV (355 nm) with the pump fluence less than 100 µJ cm^−2^. The amplified femtosecond laser was focused onto a sapphire window to create the white‐light probe pulses. The transmitted probe pulse after the 500 nm‐thick sample films (quartz substrate) was detected by a silicon photodetector after a monochromator, and the probe wavelength was selected at 570 nm (2.17 eV). The normalized transient absorption (∆*A*) was obtained by comparing the normalized changes in the transmittance of probe pulses of the samples using standard synchronous lock‐in detection. The time resolution of the measurement system was ≈280 fs. All measurements were performed at room temperature.

### Device Characterization

The electrical performance and stability of TFTs were characterized by a semiconductor parameter analyzer (Keithley 4200SCS). The transfer characteristics of the TFTs were measured with gate‐to‐source voltage (*V*_GS_) sweeping from −15 to +15 V, and *V*_DS_ fixed at 10.1 V, a voltage around which the TFTs normally work when switching/driving the pixel of the FPDs. The threshold voltage of TFTs was extracted from the value of *V*_GS_ when *I*_D_ = 10^−8^ A and the *µ*_sat_ was calculated by the following equation,

(6) $$\mu_{\mathrm{sat}}=\frac{2L}{WC_{\mathrm{ox}}}\;{\left(\frac{\partial \sqrt{I_{D}}}{\partial V_{\mathrm{GS}}}\right)}^{2}$$

where the *C*_OX_ is the unit capacitance of the gate insulator layer, and the slope term $\frac{\partial \sqrt{I_{D}}}{\partial V_{\mathrm{GS}}}$ was determined from the gradient of the linear part in the *I*_D_^1/2^ versus *V*_GS_ plot. NBS and NBIS stability were tested at *V*_GS_ = −20 V. Illumination stress was performed under a halogen light with a power density of 0.22 mW cm^−2^. The spectrum of the halogen light source is exhibited in Figure S17, Supporting Information. PBTS stability was measured at *V*_GS_ = +20 V under 60 °C in vacuum.

## Conflict of Interest

The authors declare no conflict of interest.

## Supporting information

Supporting Information

## Data Availability

The data that support the findings of this study are available from the corresponding author upon reasonable request.
