# Supplementary material for: Addressing the Conflict between Mobility and Stability in Oxide Thin‐film Transistors
Source: Adv Sci (Weinh). 2023 Mar 19;10(14):2300373. doi: 10.1002/advs.202300373 (PMC10190610; doi:10.1002/advs.202300373)
Supplement: Supplementary file 1 — Supporting Information [file ADVS-10-2300373-s001.pdf]

Supporting Information

Addressing the Conflict between Mobility and Stability in Oxide Thin-film Transistors

Lingyan Liang[1], Hengbo Zhang[1], Ting Li, Wanfa Li, Junhua Gao, Hongliang Zhang, Min Guo, Shangpeng Gao, Zirui He, Fengjuan Liu, Ce Ning, Hongtao Cao, Guangcai Yuan*, and Chuan Liu**

Prof. L.Y. Liang, H. B. Zhang, T. Li, W. F. Li, J. H. Gao, H. L. Zhang, Prof. H. T. Cao
Laboratory of Advanced Nano Materials and Devices, Ningbo Institute of Materials Technology and Engineering, Chinese Academy of Sciences, Ningbo 315201, China.
E-mail: h_cao@nimte.ac.cn
[1]These authors contributed equally: Lingyan Liang, Hengbo Zhang

Prof. H. T. Cao
Center of Materials Science and Optoelectronics Engineering, University of Chinese Academy of Sciences, Beijing 100049, China.

M. Guo, Prof. C. Liu
State Key Lab of Opto-Electronic Materials & Technologies, School of Electronics and Information Technology, Sun Yat-Sen University, Guangzhou 510275, China.
E-mail: liuchuan5@mail.sysu.edu.cn

S. P. Gao, Z. R. He
Department of Materials Science, Fudan University, Shanghai 200433, China.

F. J. Liu, C. Ning, G. C. Yuan
BOE Technology Group Co., Ltd., Beijing, China.
E-mail: yuanguangcai@boe.com.cn

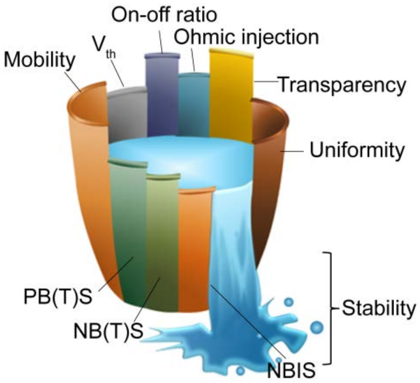

Figure S1 A schematic representation of the bucket effect for characteristics of thin-film transistors. Here, PB(T)S and NB(T)S respectively represents positive-bias (temperature) stress and negative-bias (temperature) stress.

Table S1 Effective mass for amorphous oxide semiconductors from MD calculations (the unit is free electron mass m_0).

	[1 0 0]	[1 1 0]	[1 1 1]
a-$In_{32}O_{48}$	0.278	0.281	0.280
a-$In_{24}Zn_4Sn_4O_{48}$	0.297	0.294	0.296
a-$In_{12}Zn_8Sn_{12}O_{50}$	0.295	0.298	0.290
a-$In_6Zn_{13}Sn_{13}O_{48}$	0.489	0.493	0.486

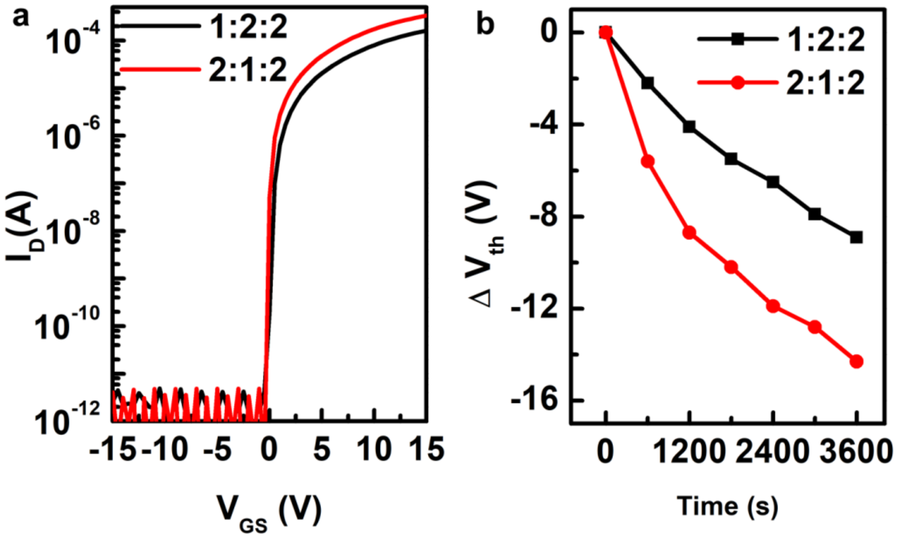

Figure S2 Transfer curves and Time-dependent NBIS ΔV_{th} of ITZO TFTs with different In:Sn:Zn ratios.

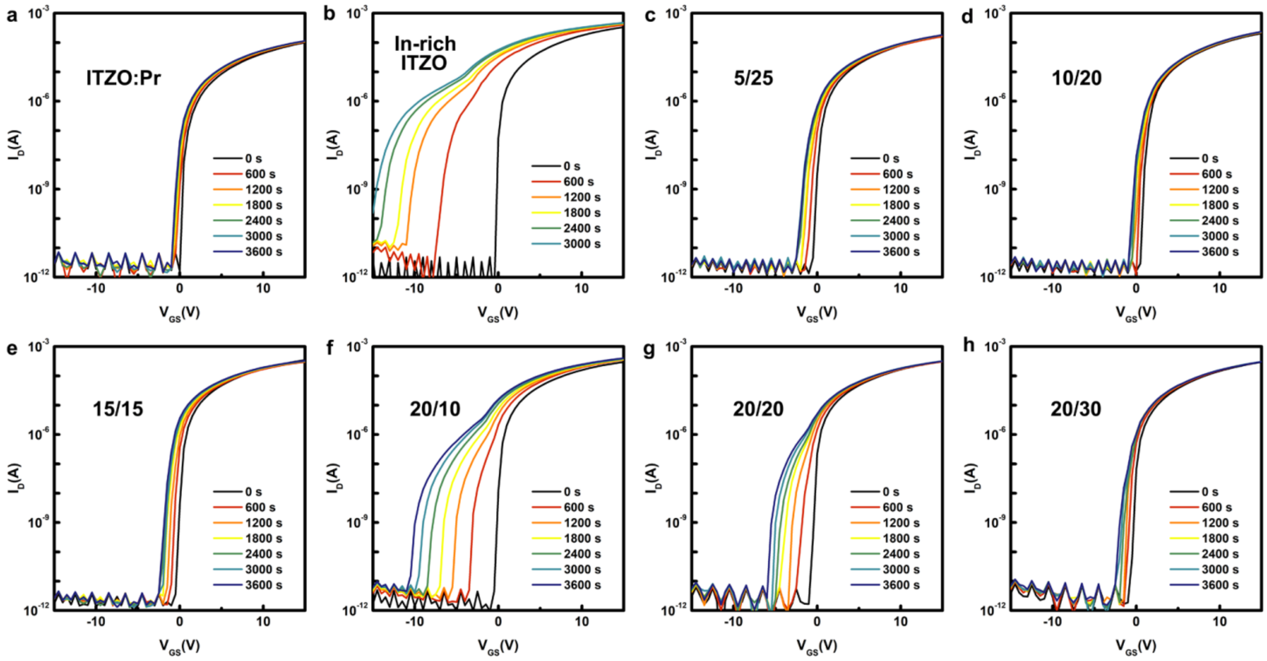

Figure S3 Time dependence of transfer curves under NBIS tests of single-layer (30 nm) In-rich ITZO (a), ITZO:Pr (b) and bilayer In-rich ITZO/ITZO:Pr TFTs (c-h, marked as the thickness ratio of CTL/CRL).

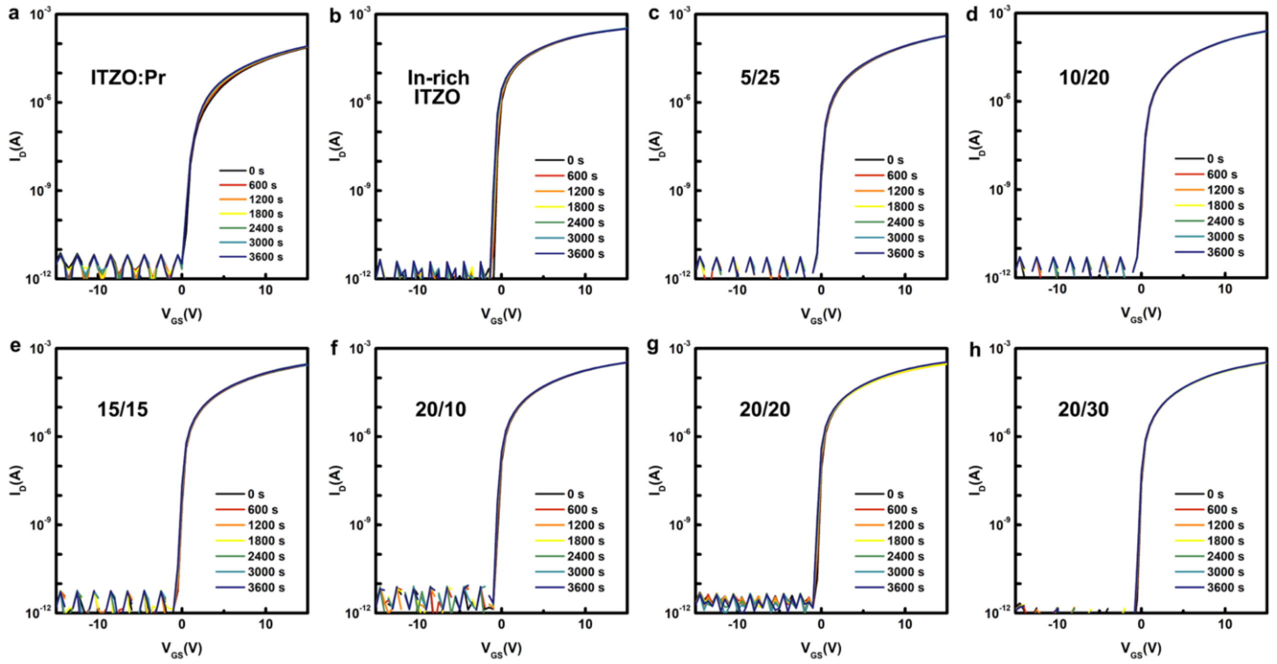

Figure S4 Time dependence of transfer curves under NBS tests (V_{GS} stress = −20 V, 3600 s) of single-layer (30 nm) In-rich ITZO (a), ITZO:Pr (b) and bilayer In-rich ITZO/ITZO:Pr TFTs (c-h, marked as the thickness ratio of CTL/CRL).

Table S2 Extracted electrical parameters of single-layer and bilayer TFTs.

Channel layer		V_{th} (V)	μ_{sat} ($cm^2\ V^{-1}\ s^{-1}$)	SS ($V\ dec^{-1}$)	I_{on}/I_{off} ($\times10^8$)	ΔV_{th} (V) NBS	NBIS	PBTS
Single-layer	ITZO:Pr	1.16	16.2	0.20	0.28	−0.10	−0.71	5.47
	In-rich ITZO	−0.19	51.6	0.15	1.31	−0.28	> −15	0.85
Bilayer (CTL/CRL)	5/25	0.22	27.2	0.22	0.85	−0.11	−1.56	2.80
	10/20	0.02	36.4	0.15	1.18	−0.07	−1.12	2.99
	15/15	−0.10	43.3	0.15	1.22	−0.13	−1.70	2.92
	20/10	−0.18	50.1	0.19	1.03	−0.21	−9.13	1.43
	20/20	−0.27	47.1	0.20	1.16	−0.20	−4.66	0.92
	20/30	−0.04	46.8	0.17	0.88	−0.18	−1.56	0.69

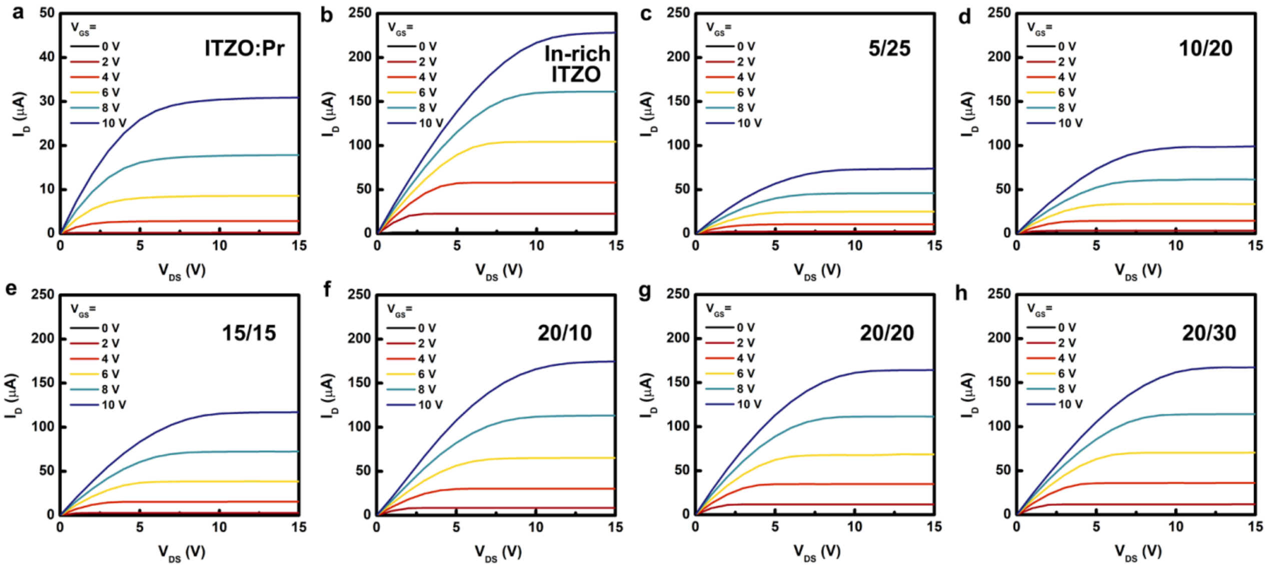

Figure S5 Representative output characteristics (I_D-V_{DS}) of single-layer (30 nm) In-rich ITZO (a), ITZO:Pr (b) and bilayer In-rich ITZO/ITZO:Pr TFTs (c-h, marked as the thickness ratio of CTL/CRL).

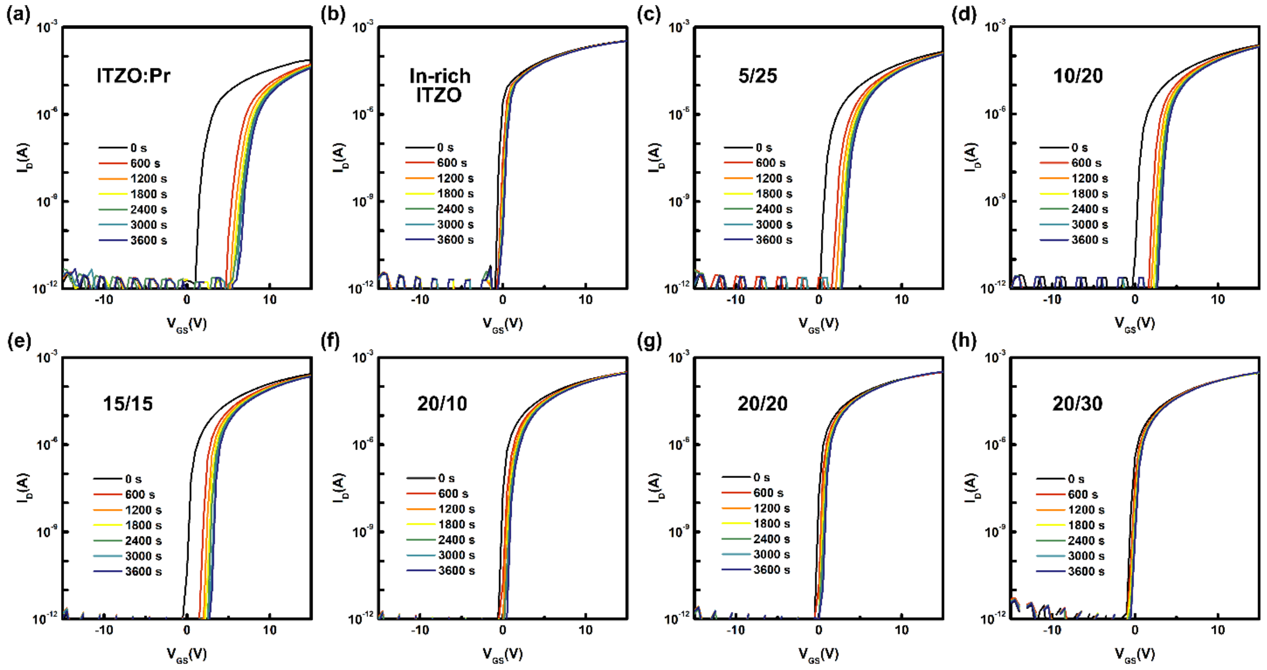

Figure S6 Time dependence of transfer curves under PBTS tests (V_{GS} stress = +20 V, 3600 s, 60 ^{o}C, in vacuum).

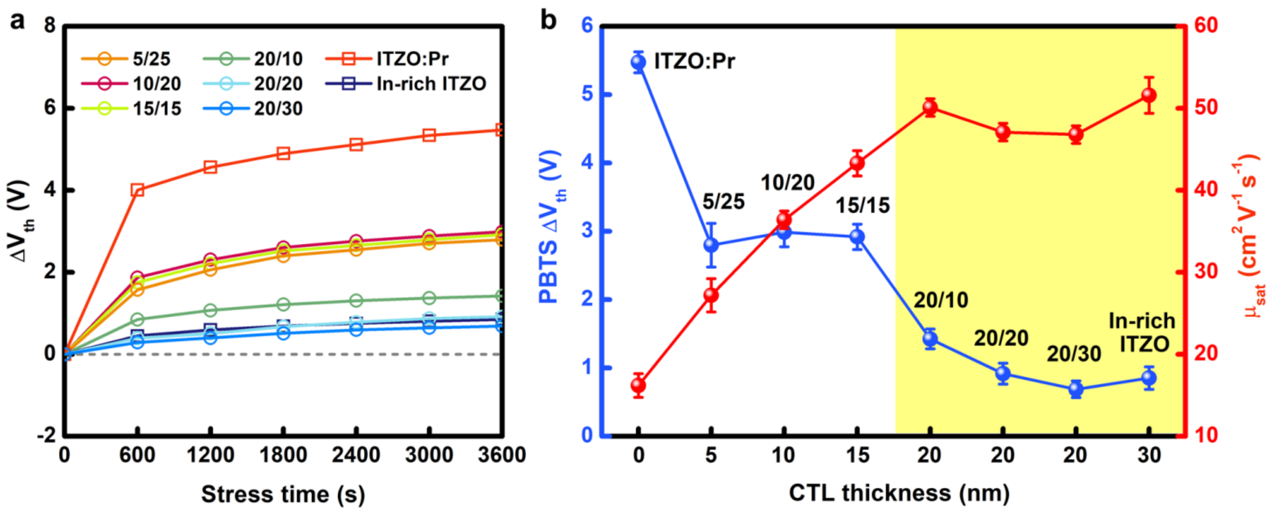

Figure S7 PBTS stability of single- and bilayer TFTs (marked as CTL/CRL). a) Time-dependent ΔV_{th} under PBTS tests. b) The PBTS ΔV_{th} and μ_{sat} extracted from the corresponding TFTs. The error bars represent the standard deviation over 15 devices. And devices with high μ_{sat} and good PBTS stability are highlighted in yellow color.

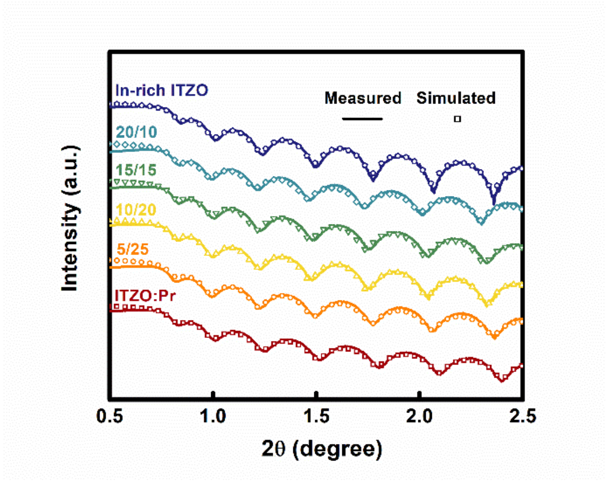

Figure S8 Measured and simulated X-ray reflectivity (XRR) spectra for AOS films.

Table S3 Surface and interface roughness and ρ_{eff} extracted from XRR spectra.

Channel layer		$R_{surface}$ (nm)	$R_{interface}$ (nm)	R_{Si} (nm)	ρ_{eff} (g cm^{-3})
Single-layer	ITZO:Pr	0.53	–	0.30	8.12
	In-rich ITZO	0.64	–	0.32	8.52
Bilayer (CTL/CRL)	5/25	0.44	0.55	0.26	7.97
	10/20	0.47	0.49	0.31	8.20
	15/15	0.42	0.55	0.28	8.27
	20/10	0.48	0.51	0.25	8.34

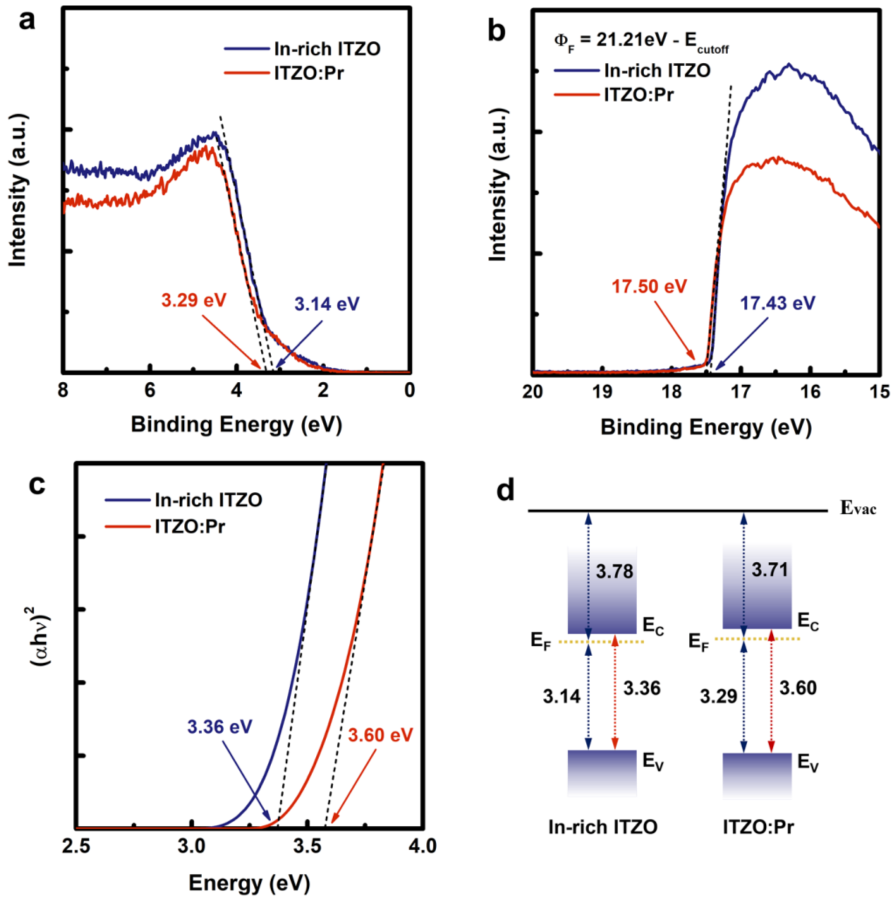

Figure S9 Valence band edge (E_{VBM}–E_F) (a) and He II spectra of secondary electron cutoff (E_{cutoff}) (b) of the In-rich ITZO and ITZO:Pr films characterized by UPS. c) The E_g values extracted from the Tauc plot (the square of the product of the absorption coefficient and photon energy versus the photon energy, i.e. $(\alpha h\nu)^2$ vs. $h\nu$)). d) Energy-level alignment diagram of In-rich ITZO and ITZO:Pr.

Table S4 Summary of fitting parameters for carrier lifetime of In-rich ITZO and ITZO:Pr.

	A_1	τ_1 (ps)	A_2	τ_2 (ps)	A_3	τ_3 (ps)
In-rich ITZO	0.31	5.5	0.49	64.5	0.20	1020.4
ITZO:Pr	0.41	3.5	0.43	39.3	0.16	740.0

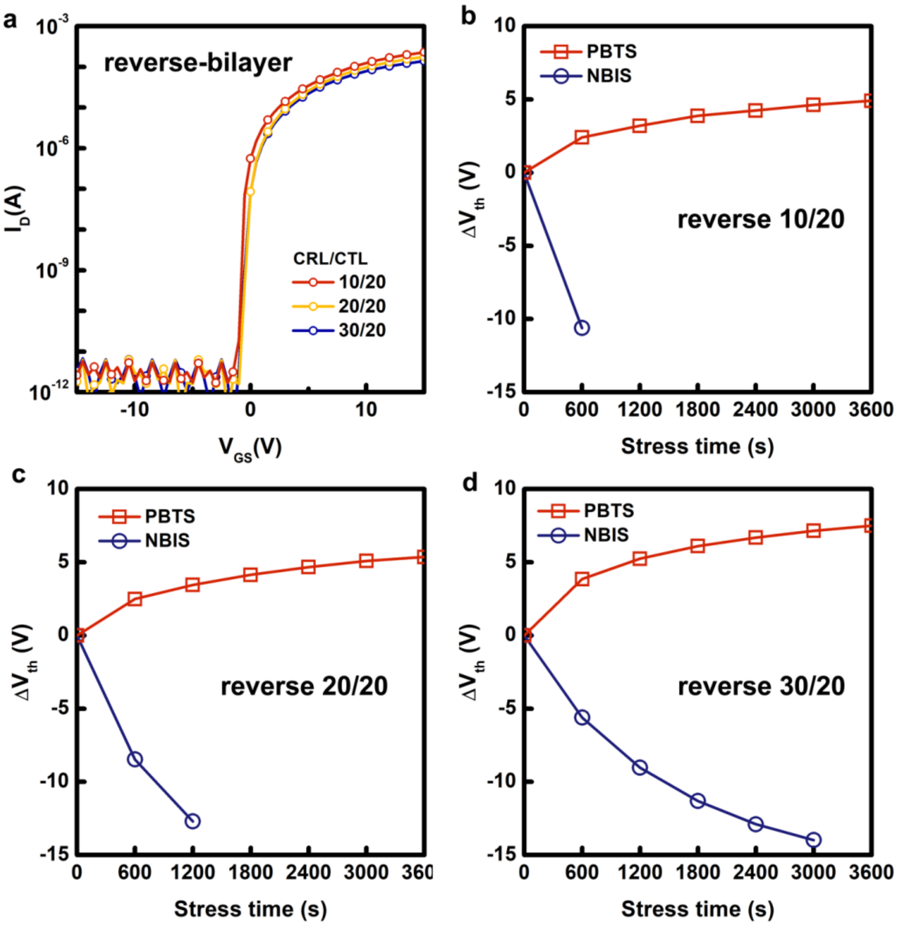

Figure S10 Electrical performance of reverse-bilayer TFTs. a) Representative transfer characteristics (I_D-V_{GS}) of reverse-bilayer ITZO:Pr/In-rich ITZO TFTs (marked as the thickness ratio of CRL/CTL). Time-dependent ΔV_{th} under PBTS and NBIS tests: reverse-bilayer 10/20 (b), reverse-bilayer 20/20 (c), and reverse-bilayer 30/20 TFTs (d).

Table S5 Extracted electrical parameters of reverse-bilayer TFTs.

Channel layer		V_{th} (V)	μ_{sat} ($cm^2\ V^{-1}\ s^{-1}$)	SS ($V\ dec^{-1}$)	I_{on}/I_{off} ($\times10^8$)	ΔV_{th} (V) NBIS	ΔV_{th} (V) PBTS
Reverse-bilayer (CRL/CTL)	10/20	−0.58	33.8	0.17	0.70	> −15	4.91
	20/20	−0.20	30.7	0.21	0.62	> −15	5.36
	30/20	−0.23	25.1	0.23	0.43	> −15	7.50

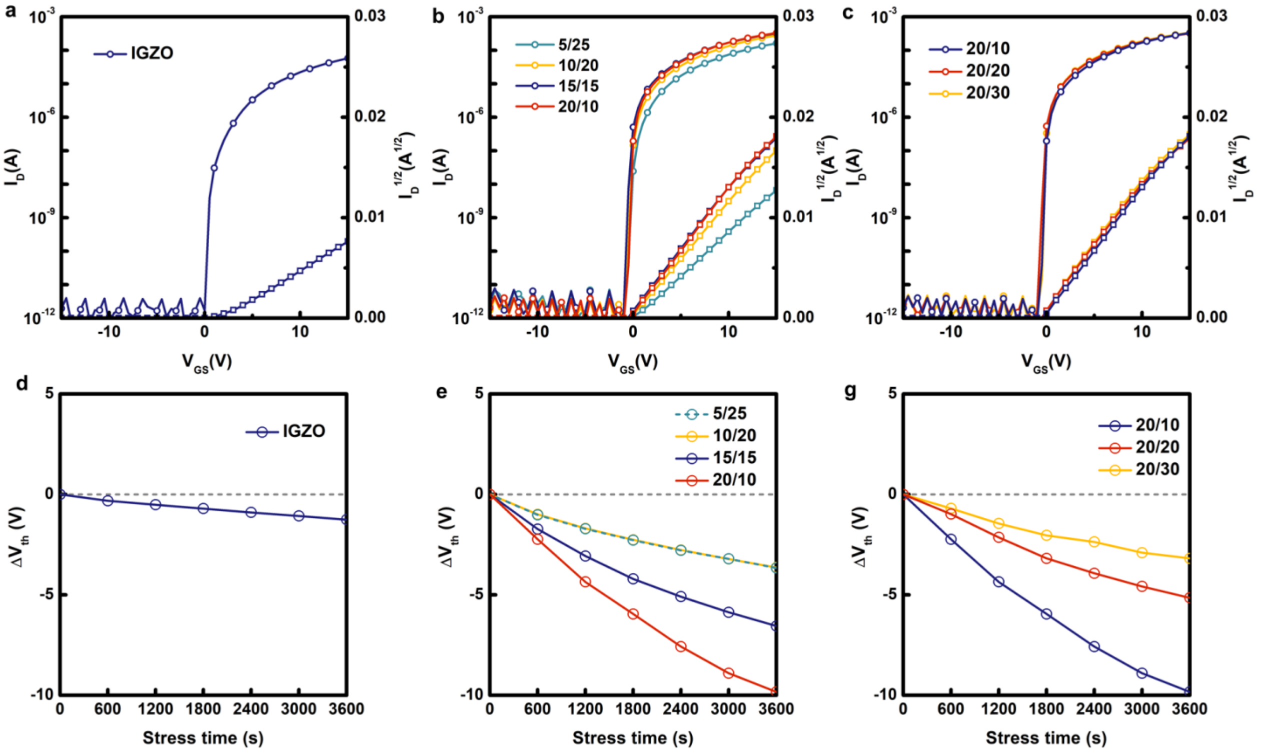

Figure S11 Electrical performance of IGZO and IGZO/ITZO TFTs. Representative transfer characteristics (I_D-V_{GS}) of single-layer (30 nm) IGZO (a) and bilayer ITZO/IGZO TFTs (b,c, marked as the thickness ratio of CTL/CRL). Time-dependent ΔV_{th} under NBIS tests: IGZO (d) and ITZO/IGZO bilayer (e, g) TFTs.

Table S6 Extracted electrical parameters of bilayer ITZO/IGZO TFTs.

Channel layer		V_{th} (V)	μ_{sat} (cm^2 V^{-1} s^{-1})	SS (V dec^{-1})	I_{on}/I_{off} (×10^8)	ΔV_{th} (V) NBS	ΔV_{th} (V) NBIS
Single-layer	IGZO	0.41	11.4	0.25	0.29	−0.14	−1.25
Bilayer (CTL/CRL)	5/25	−0.04	28.5	0.17	0.60	−0.25	−3.74
	10/20	−0.21	39.1	0.17	1.23	−0.16	−3.64
	15/15	−0.55	46.3	0.13	1.07	−0.03	−6.51
	20/10	−0.21	48.5	0.11	1.33	−0.03	−9.82
	20/20	0.01	47.8	0.15	1.49	−0.04	−5.17
	20/30	−0.10	45.5	0.14	1.30	−0.08	−3.17

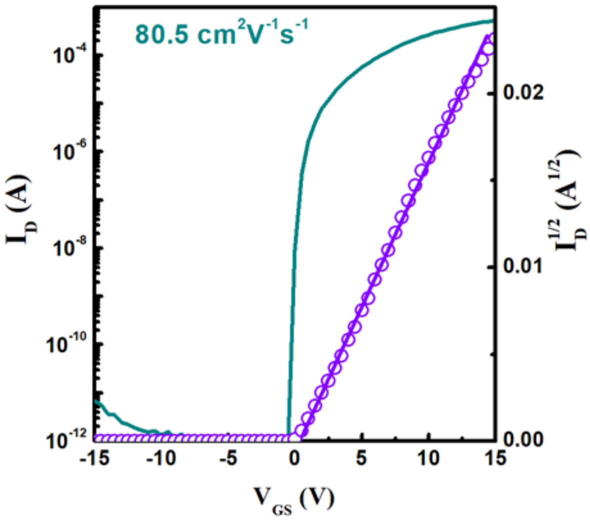

Figure S12 Transfer curves of In&Sn-rich ITZO TFTs.

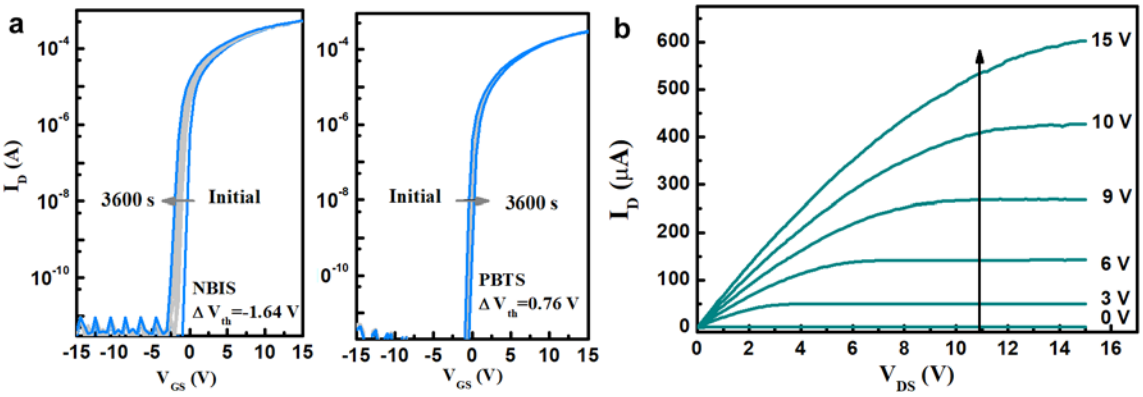

Figure S13 Time dependence of transfer curves under NBIS (a) and PBTS (b) tests, and output curves (c) of In&Sn-rich ITZO/ITZO:Pr bilayer TFTs.

Table S7 Summary of the mobility, normalized I_D and NBIS ΔV_{th} of representative AOS TFTs reported in the literatures.

Channel layer	Thickness ratio	Insulator (thickness)	Mobility ($cm^2 V^{-1} s^{-1}$)	V_{th} (V)	Normalized I_D (μA)	NBIS stability	Ref.
Oxygen-poor IGZO /IGZO	30/20	SiO_2/SiN_x (200 nm)	8.1	8.8	16	ΔV_{th} = −3.2 V (1000 s), V_{GS} stress = −20 V, white light (400-700 nm, 1000 lux)	[1]
Low-oxygen IAZO /IAZO	20/10	SiO_2 (100 nm)	12.0	4.1	30	ΔV_{th} = −0.73 V (3000 s), V_{GS} stress = −20 V, white light (1350-1600 lm)	[2]
IZO:Hf (Hf-rich) /IZO:Hf	10/40	SiN_x/SiO_x (400/50 nm)	15.0	~1.5	25 (maximum)	ΔV_{th} = −2.55 V (11000 s), V_{GS} stress = −20 V, white light (3000 cd m^{-2})	[3]
ZTO/VZTO	80/40	SiO_2 (200 nm)	16.9	7.7	45	ΔV_{th} = −0.9 V (5000 s), V_{GS} stress = −30 V, green LED (550 nm, 0.1 mW cm^{-2})	[4]
Oxygen-poor IWO /IWO	10/20	SiO_2 (100 nm)	20.4	0.52	50	ΔV_{th} = −7.2 V (2000 s), E_{stress} = −2.5 MV cm^{-1}, 60 °C, bule light (460 nm, 1.53 mW cm^{-2})	[5]
IGZO/IGZO:Hf	17/33	SiO_2 (120 nm)	31.0	~0	60	ΔV_{th} = −10 V (10000 s), V_{GS} stress = −20 V, white light (1000 lux)	[6]
IZO/ZTO	5/35	SiO_2 (120 nm)	32.3	0.5	70	ΔV_{th} = −4.1 V (7200 s), V_{GS} stress = −20 V, green LED (0.27 mW cm^{-2})	[7]
IZO/IGZO:N	15/15	SiO_2 (100 nm)	49.6	−2.3	125	ΔV_{th} = −11 V (2500 s), V_{GS} stress = −20V, UV light (380 nm, 0.1 mW cm^{-2})	[8]
IZO/AITZO	9/30	SiO_2 (100 nm)	53.2	0.5	150	ΔV_{th} = −6 V (3600 s), V_{GS} stress = −20V, white light (0.25 mW cm^{-2})	[9]
ITZO/SZO	20/5	SiO_2 (150 nm)	60/1.8 (correction factor[14])	-1	Only linear curves	ΔV_{th} = −1.33 V (3600 s), V_{GS} stress = −20V, white light (15000 lux)	[10]
ITZO	30	SiO_2 (100 nm)	30.2	-0.88	60	ΔV_{th} = −4 V (2000 s), V_{GS} stress = −25V, blue light (465 nm, 0.2 mW cm^{-2})	[11]
IGZO	50	SiO_2/SiN_x (150 nm)	25.7	−0.1	50 (maximum)	ΔV_{th} = −5 V (7200 s), V_{GS} stress = −20V, blue light (460 nm, 0.075 mW cm^{-2})	[12]
IGTO	25	SiO_2 (100 nm)	25.9 (linear)	-0.7	Only linear curves	ΔV_{th} = −4.5 V (3000 s), V_{GS} stress = −20V, white light (420-780 nm, 2000 lux)	[13]
In-rich ITZO /ITZO:Pr	20/30	SiO_2 (100 nm)	75.5	−0.04	225	ΔV_{th} = −1.64 V (3600 s), V_{GS} stress = −20V, halogen light (0.22 mW cm^{-2})	This work

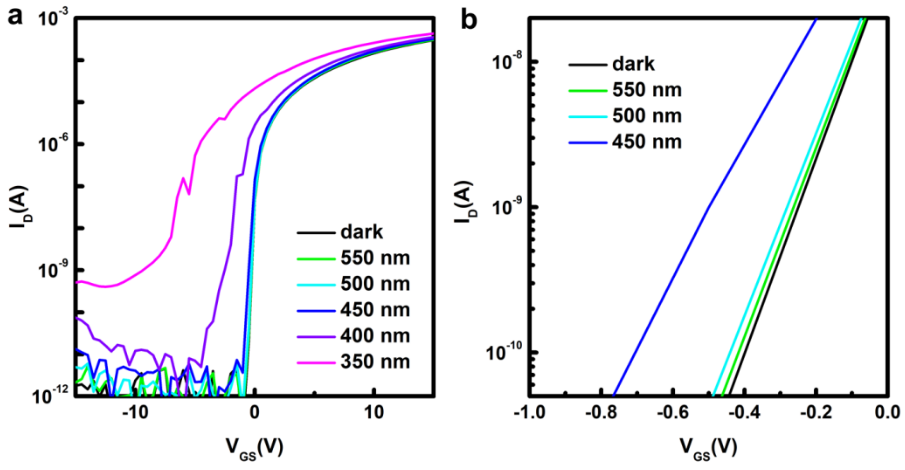

Figure S14 Monochromatic light response of the bilayer 20/30 (ITZO/ ITZO:Pr) TFTs.

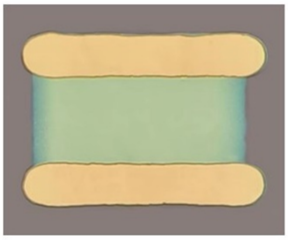

Figure S15 Top-view micrograph of the fabricated devices.

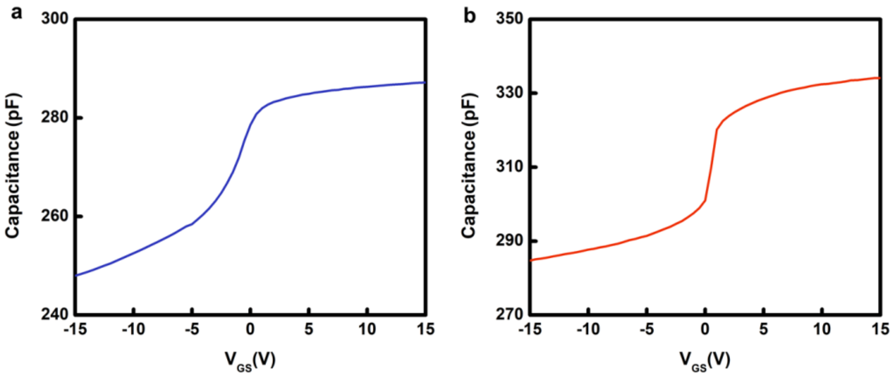

Figure S16 Capacitance-voltage (*C*-*V*) curves of the metal-insulator-semiconductor (MIS) capacitors: In-rich ITZO (a) and ITZO:Pr (b).

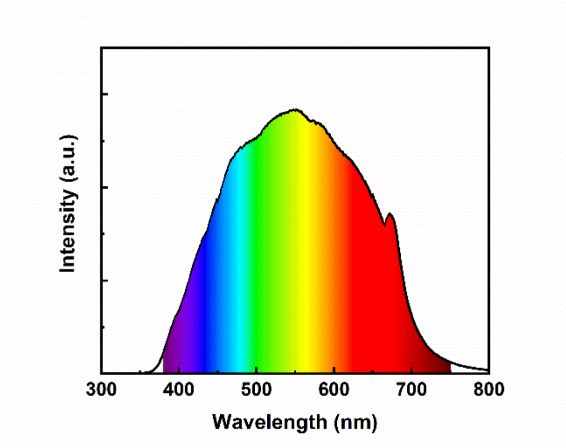

Figure S17 Spectrum of the halogen light source used in the NBIS tests.

References

[1] M. H. Kim, Y. S. Ko, H. S. Choi, S. M. Ryu, S. H. Jeon, J. H. Jung, D. K. Choi, *Phys. Status Solidi A* **2016**, 213, 1873.

[2] W. D. Xu, G. Q. Zhang, X. J. Feng, *J. Alloy. Compd.* **2021**, 862, 158030.

[3] J. C. Park, S. Kim, S. Kim, C. Kim, I. Song, Y. Park, U. I. Jung, D. H. Kim, J. S. Lee, *Adv. Mater.* **2010**, 22, 5512.

[4] M. G. Yun, C. H. Ahn, S. W. Cho, S. H. Kim, Y. K. Kim, H. K. Cho, *ACS Appl. Mater. Interfaces* **2015**, 7, 6118.

[5] P. T. Liu, C. H. Chang, C. J. Chang, *Appl. Phys. Lett.* **2016**, 108, 261603.

[6] M. S. Kim, H. T. Kim, H. Yoo, D. H. Choi, J. W. Park, T. S. Kim, J. H. Lim, H. J. Kim, *ACS Appl. Mater. Interfaces* **2021**, 13, 31816.

[7] H. Y. Jung, Y. Kang, A. Y. Hwang, C. K. Lee, S. Han, D. H. Kim, J. U. Bae, W. S. Shin, J. K. Jeong, *Sci. Rep.* **2014**, 4, 3765.

[8] H. T. Xie, Q. Wu, L. Xu, L. Zhang, G. C. Liu, C. Y. Dong, *Appl. Surf. Sci.* **2016**, 387, 237.

[9] J. H. Yang, J. H. Choi, S. H. Cho, J. E. Pi, H. O. Kim, C. S. Hwang, K. Park, S. Yoo, *IEEE Electron Device Lett.* **2018**, 39, 508.

[10] Y. S. Shiah, K. Sim, Y. H. Shi, K. Abet, S. Ueda, M. Sasase, J. Kim, H. Hosono, *Nat. Electron.* **2021**, 4, 800.

[11] P. T. Liu, C. H. Chang, C. S. Fuh, *Rsc Adv.* **2016**, 6, 106374.

[12] K. H. Ji, J. I. Kim, H. Y. Jung, S. Y. Park, R. Choi, Y. G. Mo, J. K. Jeong, *Microelectron. Eng.* **2011**, 88, 1412.
[13] D. H. Kim, H. S. Cha, H. S. Jeong, S. H. Hwang, H. I. Kwon, *Electronics* **2021**, 10, 3765.
[14] C. D. Chen, Z. H. Chen, K. J. Xu, J. W. Zheng, H. Ou, Z. G. Wang, H. J. Chen, X. Y. Liu, Q. Wu, P. K. L. Chan, C. Liu, *IEEE Electron Device Lett.* **2019**, 40, 897.
